# Effectiveness of dose-matched immersive virtual reality-stationary cycling combined with neurological physiotherapy versus neurological physiotherapy alone in individuals with chronic stroke (CIVIS): A single-blind, randomized crossover trial protocol

**DOI:** 10.1371/journal.pone.0362132

**Published:** 2026-11-06

**Authors:** Daniel Asensio-Perez, David Lucena-Anton, Jose A. Moral-Munoz

**Affiliations:** 1 Department of Nursing and Physiotherapy, University of Cadiz, Cadiz, Spain; 2 Biomedical Research and Innovation Institute of Cadiz (INiBICA), Cadiz, Spain; Università degli Studi di Napoli Federico II: Universita degli Studi di Napoli Federico II, ITALY

## Abstract

**Background:**

Stroke is a leading cause of long-term disability worldwide and frequently results in persistent motor and functional impairments, particularly affecting gait and balance. Given the high prevalence and impact of these limitations on independence and quality of life, innovative rehabilitation strategies are needed. Immersive virtual reality combined with stationary cycling (IVR-SC) has emerged as an innovative rehabilitation approach with the potential to improve mobility and functional outcomes after stroke.

**Aim:**

To analyze the comparative effectiveness of two dose-matched programs, one combining IVR-SC plus neurological physiotherapy (NP), versus the other consisting of NP alone, in individuals with chronic stroke.

**Methods:**

A randomized, single-blind, crossover clinical trial will be conducted. After calculating the sample size, a minimum of 8 participants with chronic stroke (≥6 months), hemiparesis, and gait and balance impairments will be randomly assigned to one of two sequences of intervention. Each participant will receive both neurological physiotherapy and IVR-SC across two 4-week intervention periods separated by a washout period (4 weeks). Each intervention period consists of 8 sessions delivered over a 4-week period, with each session lasting 60 minutes. The primary outcome will be gait quality, assessed using Wisconsin Gait Scale (WGS), while secondary outcomes will include walking endurance, balance, motor function, and muscle tone. Clinical outcomes will be assessed at baseline and after each intervention period. Satisfaction with using the IVR-SC intervention will also be evaluated. Statistical analyses will follow an intention-to-treat approach using a linear mixed-effects model (LMM) with treatment, period, sequence, baseline, and center as fixed effects, and participant as a random effect. Statistical significance will be set at p < 0.05. An individual responder analysis will be performed to determine if participants exceeded the minimal clinically important difference (MCID) of 2.25 points on the WGS.

**Discussion:**

This study will contribute to understanding the potential comparative effects of IVR-SC in stroke rehabilitation. Given the heterogeneity of individuals with stroke, which may limit the conclusiveness of findings in parallel-group studies, the crossover design allows each participant to serve as their own control, reducing inter-individual variability and strengthening the interpretation of the results. If effective, IVR-SC could represent a feasible and scalable option for partially replacing NP within standard neurorehabilitation, with potential for implementation in both clinical and home-based settings.

**Trial registration:**

ClinicalTrials.gov ID: NCT07529665. Effectiveness of dose-matched rehabilitation with immersive virtual reality-stationary cycling combined with neurological physiotherapy versus neurological physiotherapy alone in individuals with chronic stroke (CIVIS): study protocol for a single-blind, randomized crossover trial. URL: https://clinicaltrials.gov/study/NCT07529665. Date of registration: 2026-04-14. Version 1.

## Introduction

Stroke is a neurological syndrome caused by a cerebral circulatory disorder that results in a permanent or transient functional impairment of the affected brain areas [1,2]. Stroke accounts for approximately 11% of deaths worldwide and, although mortality related to this condition has decreased by 21% since 2000, it remains the second leading cause of disability globally [3]. The age-adjusted incidence of stroke in Europe has been estimated to range between 95 and 290 cases per 100,000 inhabitants per year, with approximately 1.1 million Europeans experiencing a stroke annually [4]. Although stroke prevalence data in Europe remain limited, the overall prevalence at the beginning of the 21^st^ century was estimated at 1.34%, corresponding to approximately six million prevalent stroke cases. Prevalence rates range from 5% in individuals younger than 75 years to more than 10% in those over 80 years of age [4].

In 2020, it was estimated that stroke incidence in Europe would increase by more than 3% by 2047, representing a 27% increase in prevalence [5]. Alongside the estimated 17% reduction in stroke-related mortality due to advances in care and treatment, this trend suggests that a greater number of individuals will survive stroke. This is expected to increase the demand for rehabilitation services and long-term care, as well as to impact quality-adjusted life years in the European population [5,6]. These trends carry substantial socioeconomic consequences: stroke accounts for approximately 1.7% of total healthcare expenditure across 32 European countries, with a total economic cost exceeding €60 billion when healthcare, social care, and productivity losses are combined [7]. In Spain, the annual cost per stroke patient has been estimated at €27,711 during the first year, representing a substantial economic burden for the healthcare system [8].

Clinical manifestations vary depending on the affected brain regions, with frequent impairments in cognitive, behavioral, perceptual, and language functions [9]. Beyond physical sequelae, stroke represents a major risk factor for mental health disorders such as anxiety and depression [9]. At the motor level, common deficits include limitations in strength, motor control, balance, and coordination [10]. The most frequent sequela is hemiparesis, characterized by sensorimotor paralysis of one side of the body [11]. Consequently, gait impairment or even the inability to walk is a common outcome following stroke [10,11]. These motor limitations, particularly alterations in gait and balance, are among the most disabling sequelae of stroke, directly compromising patients’ independence and quality of life, and representing a primary target for neurological rehabilitation [9,10].

Stroke rehabilitation aims to facilitate, maintain, or restore the highest possible level of functional capacity and independence [12,13]. Although recovery is believed to occur mainly during the first weeks after stroke, patients may continue to achieve improvements in functional tasks and experience neural reorganization several months post-stroke [14–16]. Conventional physical therapy (CPT) in stroke rehabilitation typically encompasses neurophysiological approaches such as Bobath, Perfetti, or Kabat methods [12], which are widely applied in clinical practice but frequently lack explicit specification of therapeutic content and dosage, limiting their reproducibility across studies [13,14,17]. In this study, the control intervention consists of structured neurological physiotherapy (NP), a task-oriented, multicomponent approach grounded in motor learning principles, for which strong evidence exist regarding improvements in gait speed, walking distance, balance, and lower limb motor function in stroke survivors [14,16–18].

Aerobic therapeutic exercise is gaining increasing clinical and scientific relevance as a complement to CPT in chronic stroke rehabilitation. Studies have shown that stroke survivors often exhibit poor cardiorespiratory fitness and may benefit from aerobic training [19]. Aerobic exercise protocols typically consist of 20–30 minutes per session, performed 3–5 days per week at intensities ranging from 50% to 80% of maximum heart rate (HRmax). Commonly used exercises include treadmill walking, stationary cycling (SC), and aquatic therapy [19,20]. Among these, SC has received particular attention. Accordingly, Pang et al. suggested that aerobic exercise is beneficial for improving cardiorespiratory fitness and gait performance [20], while SC specifically has been shown to improve hip and knee flexion, muscular balance between the quadriceps and hamstrings [21], and ankle proprioception in dorsiflexion and eversion control [22]. Nevertheless, SC alone does not appear to produce significant improvements in balance in individuals with chronic stroke when compared to CPT [23], suggesting the need for combined approaches that address this limitation.

Virtual reality (VR) is defined as “the simulation of a real environment generated by a computer, in which, through a human–machine interface, the user is able to interact with certain elements of the simulated scenario” [24,25]. VR is a technology with great potential for use in neurorehabilitation, as it allows the integration of CPT and neuroscience-based principles into motivating, videogame-oriented approaches [26]. Immersion, the degree to which the user perceives the virtual environment (VE) as real, is a key determinant of VR effectiveness, and depends on the presence of perceptual feedback and the level of interaction between the user and the VE [27,28]. This interaction provides feedback that allows users to evaluate the success of their actions and detect execution errors during task performance [24,29]. Immersive virtual reality (IVR), delivered through head-mounted displays, has shown promise in neurorehabilitation by enhancing engagement and enabling task-specific motor practice in ecologically valid environments [30,31].

The IVR system used in this study, HOLOFIT VR *Fitness* (HOLODIA, Strasbourg, France), is an IVR videogame that enables synchronization with external devices such as SC. Users observe their avatar from a first-person perspective cycling through different interactive VE, combining aerobic exercise with immersive sensory feedback [32]. Benefits in gait and balance have been demonstrated in individuals with stroke through repetitive exercises in VE, as well as through the combination of CPT with VR, with combined approaches being more effective than isolated interventions [33,34]. Improvements in gait speed, endurance, and symmetry have been observed both in IVR gait training interventions [33] and in SC programs [35,36]. To date, however, no study has combined IVR with SC in individuals with stroke, representing a clinically relevant gap in the literature. Motivation is also a key factor in rehabilitation, as it influences treatment adherence. Furthermore, VR has demonstrated good user acceptance and increased motivation toward therapy [37,38], which may enhance adherence, a critical factor in chronic stroke rehabilitation.

In that way, existing studies combining VR and SC [36,39,40] have employed non-IVR, limiting the potential benefits associated with full sensory immersion. Addressing this gap is particularly relevant in the chronic phase of stroke, where therapeutic options are limited and rehabilitation gains tend to plateau [14,41,42], yet functional recovery, especially in gait and balance, remains a primary demand of patients and a key determinant of independence and quality of life [9,11]. A crossover design is proposed to control the well-documented heterogeneity of stroke sequelae, allowing each participant to serve as their own control and strengthening the internal validity of the findings [43].

The primary objective will be to analyze the comparative effectiveness of two dose-matched programs, one combining IVR and SC plus NP (IVR-SC), versus the other consisting of NP alone, in individuals with chronic stroke. The secondary objectives will be: (i) to compare the effects of IVR-SC versus NP alone on gait quality, walking endurance, balance, coordination, motor function, and spasticity of the lower and upper limbs; (ii) to analyze participants’ and therapists’ perceptions and satisfaction regarding the IVR-SC intervention; and (iii) to explore associations between baseline characteristics and treatment outcomes.

## Materials and methods

### Study design

A randomized, crossover, longitudinal, and prospective clinical trial with single blinding of the evaluator is proposed. This is classified as a low-intervention clinical trial, as the exercises performed using the IVR system do not differ from those carried out in routine clinical practice, in accordance with Article 2.2.3 of Regulation (EU) No. 536/2014.This study protocol was developed and reported in accordance with the SPIRIT 2025 Statement (Standard Protocol Items: Recommendations for Interventional Trials) (S1 File).

### Study setting

The study will be conducted in multiple neurological rehabilitation centers in southern Spain, including NeuroSumma Multidisciplinary Rehabilitation Center (Málaga, Spain), DACE Sevilla Association (Sevilla, Spain), Boreas Physiotherapy Center (Cádiz, Spain), Acquired Brain Injury Association of Cádiz (Cádiz, Spain), Acquired Brain Injury Association of Jaén (Jaén, Spain), and ADACCA Day Care and Residential Center “Los Gallos” (Cádiz, Spain). These centers were selected based on accessibility to individuals with chronic stroke and their capacity to support clinical research activities.

### Participants

Inclusion criteria include adults aged 18 years or older, both male and female; a medically confirmed diagnosis of chronic ischemic or hemorrhagic stroke (≥6 months); presence of hemiparesis; mild to moderate balance impairment (Berg Balance Scale (BBS) <50); gait impairment (Wisconsin Gait Scale (WGS) >20) [44]; active movement in the lower limbs; and ability to safely mount and remain seated on a SC.

Exclusion criteria include severe cardiac conditions; visual or auditory impairments limiting the use of IVR devices; history of epilepsy; severe attention deficits or inability to cooperate with the intervention; severe cognitive impairment; and significant vestibular disorders or predisposition to dizziness or vomiting.

All eligible participants will provide written informed consent form prior to enrollment.

### Sample size

Sample size calculation was performed using G*Power software (v3.1.9.7) based on the minimal clinically important difference (MCID) for the WGS, established by Guzik et al. [45] as 2.25 points (95% CI [1.75, 2.79]; n = 50). Using a two-independent-means test (α = 0.05, power = 80%), this MCID yielded a minimum requirement of 11 participants per group. Given the crossover design, this estimate was recalculated as a paired, within-subject comparison following an adjustment factor (1 − ρ) for AB/BA crossover trials [46], assuming a conservative within-subject correlation of ρ = 0.5 in the absence of published correlation data for this outcome. This yielded a minimum of 6 participants. Considering a potential dropout rate of 20%, the multicenter nature of the study, which may introduce additional between-center variability and the heterogeneity inherent to chronic stroke sequelae, a total of 8 participants will be recruited to complete both interventions, including the corresponding washout period.

We acknowledge that anchoring the target difference to the MCID, rather than to a realistic estimate of the true expected effect, is a recognized limitation of sample size calculations [47]. The calculated sample size should therefore be interpreted as the minimum required to detect a clinically meaningful difference, rather than a guarantee of adequate power for the true underlying effect.

### Allocation and randomization

Participants will be randomly allocated to one of the two intervention sequences using Epidat 4.2 software. Using its module for subject assignment to treatments with equal-sized groups, two lists of 30 non-repeating random numbers between 1 and 60 will be generated. A member of the research team (J.A.M-M.) will generate the randomization lists prior to enrollment, will be responsible for assigning participants to intervention sequences and will distribute them to the clinical supervisors from each participating clinical center, ensuring that the outcome assessor remains unaware of individual participant allocation throughout the study. If the initially estimated sample size is exceeded, a balanced groups strategy will be applied, in which the probability of assignment to each group will be inversely proportional to the number of participants already allocated.

In this crossover design, all participants will receive both interventions across two intervention periods separated by a 4-week washout period. Group A will receive NP during the first period, followed by IVR-SC after washout. Group B will receive IVR-SC during the first period, followed by NP alone after washout.

A 4-week washout period was selected based on comparable crossover trials in stroke rehabilitation, which have employed similar washout intervals between intervention periods [48–50]. We acknowledge, in line with Patten et al. [50], that carry-over effects are of particular concern in exercise-based interventions, where the explicit intent is to induce persistent physiological and motor-learning changes, and that in the worst case, if treatment effects are retained through the washout period, a crossover comparison could simply demonstrate that more therapy is beneficial regardless of type. Furthermore, a crossover design may indicate order effects that would maximize the structure of rehabilitative activities and reveal differential impacts of intervention. To address this risk, carryover, period, and sequence effects will be formally tested prior to the primary effectiveness analysis, as described in the Statistical analysis section.

### Training

Prior to the baseline assessment, videoconference meetings will be held with the clinical supervisors of each collaborating center to explain in detail the intervention protocol for both groups and to resolve any questions before study initiation. Additionally, the S2 File will be provided, and instructions will be given on how to record maximum heart rate (HRmax) and pedaling cadence using the cadence sensor, which are the only variables collected daily by the centers.

A video tutorial will also be provided demonstrating how to download and use the HOLOFIT VR software in the different modalities described in the intervention protocol. Finally, a videoconference session will be conducted in which the clinical supervisor from each center will simulate the intervention and assessment procedures to verify that the training process has been successfully completed.

### Blinding

A single-blind, outcome-assessor-blinded, design will be implemented, in accordance with methodological recommendations for clinical trials involving immersive technology in neurorehabilitation [41]. Participant blinding is not possible, as participants are necessarily aware of the intervention they receive. This is an inherent limitation of exercise-based and IVR clinical trials and does not reflect any fault of the investigative team. However, this does not compromise the internal validity of the study as long as outcome assessors remain blinded [41].

The randomization sequence will be generated and securely maintained by a member of the research team (J.A.M-M.) who is not involved in participant recruitment, intervention delivery, or outcome assessment. After baseline assessment, the clinical supervisor at each center will receive the assigned intervention sequence and will organize treatment delivery. These supervisors will be responsible for identifying, screening, and recruiting potentially eligible participants. Treating physiotherapists will have access only to the information required to deliver the assigned intervention.

The assessor (D.A.-P.) will work exclusively with coded data. The correspondence between codes and participant identity will only be accessible to the clinical coordinators at each center, ensuring allocation concealment during assessments. Participants and treating physiotherapists will be reminded before each assessment not to disclose the intervention received. Any inadvertent unblinding will be documented, including its timing and reason.

The primary data analysis will be performed using treatment labels masked as A and B until the primary analysis has been completed and the analysis dataset has been locked.

### Data collection and data management

The clinical supervisor at each center will be responsible for participant recruitment and for inviting individuals who have experienced a stroke and meet the previously described inclusion and exclusion criteria to take part in the study.

Participants will undergo the baseline assessment, which will include clinical outcome measures and the collection of sociodemographic data. This baseline assessment will be conducted within the two weeks preceding the start of the intervention. After completion of the first intervention period (phase 1), the first post-intervention assessment will be performed within a maximum period of two weeks following the intervention. After completion of the second intervention period (phase 2), the final post-intervention assessment will likewise be conducted within two weeks of intervention completion. All assessments will be carried out by the principal investigator. The schedule of enrollment, interventions, and assessments is presented in Fig 1.

**Fig 1:**
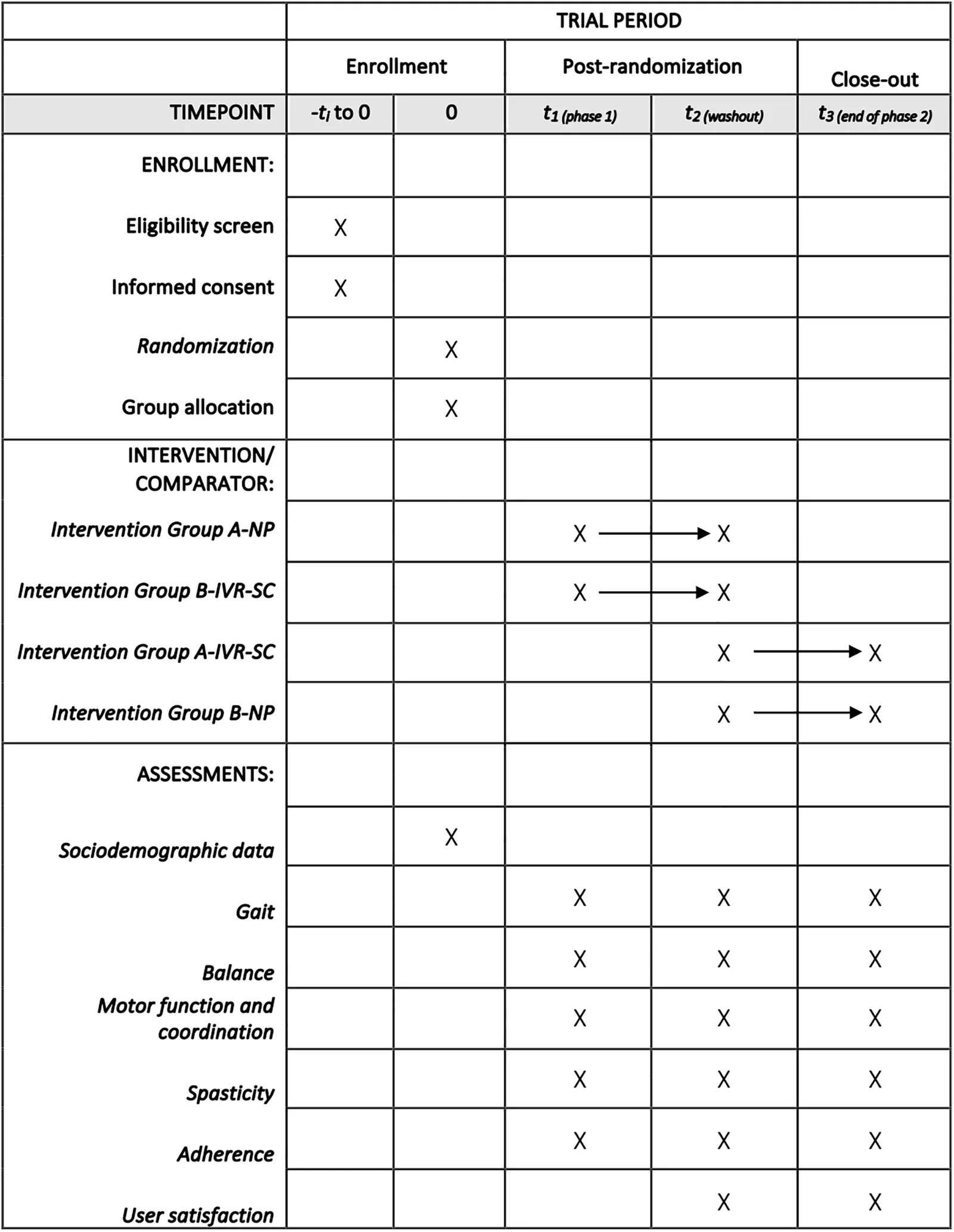
SPIRIT diagram of the schedule of enrolment, interventions and assessments.

Participant recruitment is expected to be completed by February 2027, with data collection due to finish in May 2027. We anticipate that the results will be ready for publication in June 2027.

All data will be recorded in the Case Report Form (S3 File) and subsequently entered a secure electronic database. Data entry will be performed by the principal investigator (D.A.-P.) and verified through a double-entry process conducted at different time points, with discrepancies cross-checked against the original source data. To uphold participant confidentiality, all study data will be anonymized before analysis by removing direct identifiers and coding indirect variables, in compliance with applicable ethical and data protection regulations. No datasets were generated or analyzed during the current study. All relevant data from this study will be made available upon study completion. The database will be password-protected, and access will be restricted to authorized members of the research team. Regular backups will be performed to ensure data integrity.

### Outcomes

Assessments will be conducted at baseline, during the washout period, and after the second intervention period. The following outcomes will be evaluated using their respective measurement instruments:

#### Primary outcome.

##### Gait quality:

The primary outcome will be gait quality, assessed using the WGS for qualitative evaluation. The WGS consists of 14 observable items that analyze gait components. Each item is scored from 1 (normal) to 3 (atypical), except for item 1 (scored from 1 to 5) and item 11 (scored from 1 to 4). Total scores range from 13.35 to 42, with higher scores indicating greater gait impairment. WGS scores of 24.975 at six months post-stroke and 21.65 at twelve months have been associated with a moderate risk of falls. The validity of the WGS and its correlation with other gait and balance assessment tools, such as the BBS, have been previously demonstrated [44,51].

#### Secondary outcomes.

##### Walking endurance:

Walking endurance will be assessed using the Six Minutes Walk Test (6MWT). The 6MWT measures the total distance (in meters) that the participant can walk within six minutes. The test is performed on a 30-meter course marked by two cones, with a chair available for rest if needed. The total distance covered is recorded, and any assistive devices used during the test are documented [52].

##### Balance:

Balance will be assessed using the BBS. This scale consists of 14 items that evaluate static and dynamic balance in standing position, as well as the ability to perform transfers and turning tasks. Scores range from 0 to 56, with lower scores indicating greater balance impairment. The validity of the BBS in individuals with stroke has been established [53].

##### Motor function and coordination:

Motor function will be assessed using the lower extremity subscale of the Fugl-Meyer Assessment (FMA-LE). The FMA-LE is a standardized tool for evaluating post-stroke recovery and includes five domains. In this study, the motor function and coordination/speed domains of the lower extremity will be evaluated. Each item is scored on a 3-point ordinal scale (0 = cannot perform; 1 = performs partially; 2 = performs fully), with a maximum score of 34 points for the selected domains. This measure allows the evaluation of lower limb motor function and coordination [54,55].

##### Spasticity

Spasticity will be assessed using the Modified Ashworth Scale (MAS), the most widely used standardized clinical scale for evaluating muscle tone and spasticity in rehabilitation settings [56]. The MAS measures resistance to passive muscle stretching on a 0–4 scale, where 0 indicates no increase in muscle tone, and 4 indicates rigidity in flexion or extension. Spasticity will be evaluated in the hip adductors, hamstrings, and triceps surae of the lower limb, as well as in the pectoralis major, biceps brachii, and finger flexors of the upper limb.

##### User satisfaction and perceived improvement:

To assess satisfaction with IVR as a therapeutic tool, both participants and clinicians will rate their satisfaction with the intervention program and technological devices on a 0–10 numeric scale, where 0 indicates very low satisfaction and 10 indicates complete satisfaction. Perceived improvement will be assessed using the Patient Global Impression of Improvement (PGI-I) questionnaire, a single-item measure in which participants rate their perceived change in condition following the intervention [57].

##### Sociodemographic and clinical data:

Sociodemographic and clinical data will be collected using a structured Case Report Form, including age, sex, family history of stroke, height, weight, body mass index (BMI), Charlson Comorbidity Index (CCI), date of stroke, time since stroke onset, brain lesion location, stroke type, laterality, Montreal Cognitive Assessment (MoCA), Modified Rankin Scale (mRS) [58], frequency and duration of physiotherapy sessions per week, use of assistive devices for ambulation, and main sequelae. Attendance to intervention sessions will be recorded, as well as the occurrence of cybersickness (IVR-related discomfort) [31] during sessions in the experimental group. All information will be documented in the Case Report Forms (S3 File).

### Intervention

Both Groups A and B will undergo a total of 16 dose-matched sessions, each lasting 60 minutes, delivered over an 8-week period at a frequency of two sessions per week. Specifically, each intervention period (pre-washout and post-washout) will include eight sessions conducted over four weeks, with a 4-week washout period established between both intervention periods.

During the first intervention period, Group A will receive 60-minute sessions of NP, structured into six therapeutic objective blocks. These blocks are described in Table 1. Each block will be allocated approximately 10 minutes per session, depending on the individual needs of the participants.

**Table 1:** NP intervention therapeutic blocks.

Gait training	Gait rehabilitation will be addressed through different training modalities, including obstacle walking, tandem gait, dual-task walking involving number summation, weight-shifting exercises toward the affected lower limb in standing position, and pelvic dissociation exercises.
Trunk control	From both sitting and standing positions, participants will perform reaching tasks toward objects placed in different directions, abdominal strengthening exercises, and posture-oriented activities aimed at improving postural control. Postural re-education will also be emphasized.
Balance, righting and stepping reactions	From sitting and standing positions, postural reactions will be trained through light perturbations applied in multiple directions and at varying intensities. Participants will also be instructed to generate voluntary perturbations and maintain balance before returning to the initial position.
Lower limb strengthening	Lower limb strengthening will include exercises such as glute bridges, lunges, clamshell exercises, squats, and step-up and step-down tasks.
Stretching of the affected lower limb	Intervention will focus on both passive and active stretching of the triceps surae, hamstrings, hip adductors, and iliopsoas muscles.
Foot sensory stimulation	Finally, tactile and proprioceptive sensitivity will be addressed using different textures and localization recognition exercises to normalize foot sensory function.

Group B will receive an IVR-SC intervention. This intervention will consist of 30-minute IVR sessions using the Meta Quest 2 and Meta Quest 3 VR headsets and the HOLOFIT Premium VR software (server license) combined with SC (stationary bicycles available at each neurological rehabilitation center), plus 30 minutes of structured NP. Exercise intensity will be progressively increased across the five intervention phases described in Table 2, based on each participant's tolerance, fatigue level, and ability to maintain heart rate within the prescribed training zone. In this case, due to the reduced time allocated to NP, each therapeutic block will be assigned approximately 5 minutes, depending on the individual needs of the participant.

**Table 2:** IVR-SC intervention protocol.

***1. Exploration*** *10min*	Low-intensity phase	50% – 70% HRMax
	High-intensity phase	70% – 80% HRMax
***2. Cardiogoals*** *5min*	Number of repetitions adjusted to complete the required time 70% – 80% HRMax	
***3. Rest period*** *Max. 5 min*	According to participant´s needs	
***4. Race (1 km)*** *5min*	75% – 90% HRMax	
***5. Exploration*** *5min*	50% – 70% HRMax	

The “HOLOFIT VR Fitness” software is an IVR platform that enables physical activity in a wide variety of engaging and interactive VE, while also recording exercise performance data. The interaction between the IVR device and the SC system is achieved through a cadence sensor (Magene S3+ cadence sensors).

Three exercise modalities are available: (i) exploration: participants move through different themed VE. Movement is guided and linear, and steering control is not required. The environment includes interactive elements such as greetings from virtual characters and animal encounters. It also incorporates visual engagement tasks, such as identifying and maintaining gaze on specific marked objects within the environment to promote interaction and attentional engagement. An internal radio with different music styles is available; (ii) Cardiogoals: this mode includes five difficulty levels and various time-duration options (from 5 minutes to 1 hour). During navigation through the VE, participants encounter arch-shaped panels indicating the target speed required to pass through them. A visual bar signals whether the participant is cycling too slowly or too fast (red zones at the extremes) or within the required range (green central zone). Higher difficulty levels require greater cycling speed; and (iii) race: this mode includes five difficulty levels and various race distances (from 500 meters to 21 kilometers), allowing either short, high-intensity efforts or longer endurance efforts. Participants compete against virtual avatars controlled by the game’s artificial intelligence. During each session, participants will pedal while viewing their avatar cycling in first-person perspective within the selected VE. The system allows tracking of performance scores and supports competitive dynamics between users to enhance therapeutic motivation [25].

The experimental intervention (IVR-SC) will consist of five sequential blocks completed within each session, incorporating progressively increasing cycling intensity, and the three IVR modalities (Exploration, Cardiogoals, and Race). The complete protocol, including block structure, fixed durations, and intensity level, is described in Table 2, resulting in a total IVR-SC duration of 30 minutes per session. Although block durations are predefined, minor adjustments may be made based on individual fatigue levels and functional capacity, given the inherent variability of chronic stroke sequelae.

Exercise intensity will change across session phases according to heart rate (HR), measured using a pulse oximeter. SC resistance will be adjusted to maintain the participant’s HR within the target percentage range of maximum heart rate specified for each phase. HRmax will be calculated using the Tanaka formula (208 − 0.7 × age) [59]. A monitoring document will be prepared for each participant, including HRmax values and corresponding training zones to ensure continuous supervision and safety during intervention sessions (S2 File). The same document will also record cycling resistance levels and pedaling cadence (revolutions per minute) required to reach the different HRmax percentages. These parameters will be determined during a training session with the IVR system. Attendance to sessions and the occurrence of cybersickness during intervention periods will also be documented by the clinical supervisor (S2 File).

Before starting the IVR-based intervention, the research team will conduct a training session with the IVR system. During this session, participants will experience the visual motion of the device to promote adaptation and to identify possible symptoms of cybersickness before beginning the intervention. The clinical supervisor at each center will be responsible for delivering interventions for both Group A and Group B.

During the second intervention period, after the washout phase, Group A will receive the IVR-SC intervention, and Group B will receive NP intervention.

### Statistical analysis

Statistical analyses will be performed using Jamovi version 2.7.38 software (The jamovi project, https://www.jamovi.org) with the GAMLj module for generalized and mixed linear models (Gallucci M. GAMLj: General Analyses for Linear Models in Jamovi, https://gamlj.github.io/), following an intention-to-treat approach, whereby all randomized participants will be analyzed in the group to which they were allocated, regardless of protocol adherence or dropout. A per-protocol analysis will additionally be conducted as a sensitivity analysis. The same mixed-effects model will be applied, and it will be used only to assess the robustness of the primary intention-to-treat findings. A descriptive analysis will summarize qualitative variables using absolute frequencies (n) and relative frequencies (%), and quantitative variables using measures of central tendency (mean) and dispersion (standard deviation). Both treatment groups will be compared at baseline in terms of demographic and anthropometric characteristics. The Shapiro-Wilk test will assess the normality of quantitative variables.

For each primary and secondary outcome, a linear mixed-effects model (LMM) will be fitted for normally distributed outcomes, or a generalized linear mixed model (GLMM) for non-normally distributed outcomes. Treatment, period, sequence, baseline value, and center will be entered as fixed effects, with participant included as a random effect to account for the crossover structure. The primary outcome will be gait quality measured via the WGS. The primary endpoint and time point for primary and secondary outcomes will be the mean change in the WGS total score from baseline to week 4 within each intervention period. The treatment contrast will be the estimated difference in WGS score change between IVR-SC and NP interventions. This corresponds to the standard analysis-of-variance approach recommended for AB/BA crossover trials [46]. A non-significant sequence term will be interpreted as an absence of evidence for a carryover effect. If a significant sequence effect is observed, the treatment effect estimate will be interpreted with caution, and, following the two-stage logic described by Grizzle [60], a sensitivity analysis restricted to first-period data only will be conducted. Standardized coefficients (β) derived from the model parameters will be reported as the effect size measure. Statistical significance will be set at p < 0.05.

An individual responder analysis will be conducted for the WGS. For each intervention period, participants will be classified as responders if their total WGS score decreases by at least 2.25 points, as determined by the MCID [45]. The number and percentage of responders will be reported.

### Safety considerations

Participants will be monitored throughout each session for any adverse events, including symptoms of cybersickness (e.g., dizziness, nausea) or excessive fatigue. Heart rate will be continuously monitored using a pulse oximeter to ensure exercise intensity remains within the prescribed training zones. A session will be immediately paused or terminated if any of the following occur: chest pain, dyspnea, dizziness, or any other adverse symptoms reported by the participant. Cybersickness symptoms will be recorded after each IVR-SC session using a standardized registry. If a participant reports significant cybersickness symptoms in more than one consecutive session, the clinical supervisor will evaluate whether intervention should be modified or discontinued. In case of emergency, the centers are equipped with medical personnel and specialized tools to treat or prevent injuries and health issues. All adverse events will be recorded in the Case Report Form (S3 File) and reported to the principal investigator.

### Ethical considerations

The study has been approved by the Coordinating Committee of Biomedical Research Ethics of Andalusia with Code SICEIA-2025–003245 held on December 17, 2025 (Minutes 11/25). The protocol complies with the principles of the Declaration of Helsinki and with Good Clinical Practice standards.

All participants will receive verbal and written information about the study and will sign the corresponding informed consent form prior to inclusion. Participation will be voluntary, and participants will have the right to withdraw at any time without consequences for their clinical care. Informed consent forms will be securely stored by the clinical supervisor at each participating center, ensuring confidentiality and compliance with applicable data protection regulations. Personal data will be processed in accordance with Regulation (EU) 2016/679 (General Data Protection Regulation) and Spanish Organic Law 3/2018. Data will be treated confidentially and anonymized prior to analysis.

### Dissemination policy

Trial results will be published in peer-reviewed journals regardless of the direction or magnitude of the findings, with authorship determined according to International Committee of Medical Journal Editors (ICMJE) criteria. Findings will also be presented at relevant scientific conferences and communicated to participants and collaborating centers. The ClinicalTrials.gov registry will be updated upon study completion.

## Discussion

The present study presents a novel dose-matched protocol comparing a combined IVR-SC program within NP versus NP alone in individuals with chronic stroke. While both therapies have demonstrated independent benefits in individuals with stroke [23,34,41], their combined effect using IVR has not been previously investigated [33,36,39,40]. The crossover design further strengthens the methodological approach by controlling the inter-individual variability inherent to stroke sequelae, allowing more robust within-subject comparisons [14,42,43].

The combination of IVR and CPT has been shown to improve balance and coordination in individuals with stroke [33,34], which provides a rationale for integrating IVR into a structured NP program. This is particularly pertinent given that NP incorporates the same functional domains targeted in those trials, namely gait training, balance, and lower limb motor function [13,14]. Therefore, it is reasonable to suggest that delivering IVR within this more clearly defined therapeutic framework could lead to similar or better results. Similarly, the repetitive and cyclical nature of SC may contribute to improvements in muscle tone and lower limb strength [23]. From a functional perspective, the incorporation of the aerobic component associated with SC is expected to improve walking endurance [20], while the integration of immersive VEs into therapy may enhance gait quality, including parameters such as step length, symmetry, and speed. Previous studies using similar devices support this physiological and functional plausibility [33–36].

Beyond physical outcomes, the motivational component of IVR should also be considered. The use of interactive and gamified environments has been associated with increased adherence and patient engagement in therapy [37,38]. In the present study, this dimension will be specifically assessed through a 0–10 numeric satisfaction scale completed by both participants and clinicians, as well as the PGI-I questionnaire, providing quantitative data on user acceptance and perceived therapeutic benefit that are often lacking in similar trials.

If favorable results are obtained, this study may provide preliminary evidence supporting the clinical application of a combined intervention based on IVR and aerobic exercise through SC in individuals with chronic stroke. This would allow the development of more specific protocols exploring different software platforms, exercise modalities, and interaction formats, including the competitive multi-user mode offered by HOLOFIT, with the aim of analyzing its impact on treatment adherence and clinical outcomes. Conversely, if the expected effects are not observed, it will be necessary to determine whether the proposed intervention presents limitations in its design or whether the role of IVR in neurorehabilitation has been overestimated in relation to its real clinical benefits compared with standardized NP.

Despite its strengths, the results of this study should be interpreted considering several limitations. First, although crossover trials can minimize the impact of confounding variables and individual variability by enabling participants to act as their own baseline controls, thereby offering greater statistical power and allowing for a smaller sample size [50], the selection of an adequate washout period is critical in crossover trials. Insufficient washout periods are a common limitation, and the adequacy of washout periods is rarely justified in crossover trials [61]. To address this issue, the effects of carryover, period and sequence will be formally tested prior to the primary effectiveness analysis. Second, although outcome assessors will be blinded, the nature of the intervention prevents blinding of participants and therapists, which may introduce expectation bias. Third, the adaptability of the duration and intensity of the IVR-SC intervention according to individual fatigue and functional capacity may limit strict protocol standardization due to variability in stroke severity. Fourth, although block durations are predefined, minor adjustments may be made to address this variability, a certain degree of heterogeneity is expected. Fifth, the absence of follow-up assessments at three or six months prevents evaluation of the long-term effects of the intervention. Future studies should examine the persistence of potential benefits over time. Sixth, some participants may experience difficulties in handling immersive technologies; to mitigate this, a training session will be conducted prior to the intervention, and technical support will be provided throughout the study. Finally, findings from this study may not be generalizable to other stroke populations with different clinical profiles, severity levels, or rehabilitation contexts, given the inherent heterogeneity of chronic sequelae [42]. Findings should therefore be interpreted with caution and replicated in larger and more diverse samples.

## Supporting information

S1 File Spirit Checklist 2025. (DOCX)

S2 File Clinical Supervision Documentation. (XLSX)

S3 File Case Report Forms. (PDF)
