# Supplementary material for: Effectiveness of dose-matched immersive virtual reality-stationary cycling combined with neurological physiotherapy versus neurological physiotherapy alone in individuals with chronic stroke (CIVIS): A single-blind, randomized crossover trial protocol
Source: PLoS One. 2026 Nov 6;21(11):e0362132. doi: 10.1371/journal.pone.0362132 (PMC13742934; doi:10.1371/journal.pone.0362132)
Supplement: Supplementary file 1 [file pone.0362132.s003.pdf]

UCA Universidad de Cádiz

EFFICACY OF IMMERSIVE VIRTUAL REALITY COMBINED WITH STATIONARY CYCLING IN INDIVIDUALS WITH STROKE (CIVIS): STUDY PROTOCOL FOR A RANDOMIZED CONTROLLED TRIAL

IDD CODE: ____________ **EVALUATOR:** ______________________ **ASSESSMENT DATE:** ____ /____ /______

PERSONAL DATA

DATE OF BIRTH: ____ /____ /______ **AGE:** ______ years **SEX:** Female ☐(0) Male☐(1)

CONTACT PHONE: ________________________ **PROVINCE OF RESIDENCE:** ___________________________________

FAMILY HISTORY OF STROKE: No ☐(0) Yes ☐(1)

CLINICAL DATA

- TIME SINCE STROKE (months): ____________________ **DATE OF STROKE:** ___ /___ /_____

- AFFECTED BRAIN REGION: ____

- STROKE TYPE: ISCHEMIC ☐(0) HEMORRHAGIC ☐(1)

- HEMIPARESIS LATERALITY: RIGHT ☐(0) LEFT ☐(1) BILATERAL ☐(2)

- PHYSIOTHERAPY SESSION FREQUENCY (ss/w): ________ **WEEKLY THERAPY DURATION (h/w):** _______

- USE OF ASSISTIVE DEVICE FOR MOBILITY: No ☐(0) Yes ☐(1)

☐ None ☐ Wheelchair ☐Cane ☐ Crutch ☐ Tripod Cane ☐ Walker
☐ Ankle-Foot Orthosis ☐ Other. Specify: ____

- CURRENT STROKE-RELATED IMPAIRMENTS:

	**Comments**		**Comments**
☐Hemiparesis	____________		
☐ Ataxia	____________		
☐ Aphasia	____________	☐ Spasticity	____________
☐ Neglect	____________	☐ Frozen Shoulder / Periarthritis	____________
☐ Depression	____________	☐ Stroke-Related Pain	____________
________	____________	________	____________
________	____________	________	____________

CLINICAL ASSESSMENTS

CHARLSON COMORBIDITY INDEX (CCI)

Age: ____________

Myocardial Infarction:		Diabetes:	
Congestive Heart Failure:		Diabetes with End-Organ Damage:	
Peripheral Vascular Disease:		Hemiplegia:	
Cerebrovascular Disease:		Moderate or Severe Renal Disease:	
Dementia:		Any Tumor:	
Chronic Pulmonary Disease:		Leukemia:	
Connective Tissue Disease:		Lymphoma:	
Peptic Ulcer Disease:		Metastatic Solid Tumor:	
Mild Liver Disease:		AIDS:	
Moderate or Severe Liver Disease:			X

CCI Score: ____________

Estimated 10-Year Survival: ____________%

MODIFIED RANKIN SCALE (MRS) (0-6)

	0. No Symptoms
	1. No Significant Disability
	2. Slight Disability
	3. Moderate Disability
	4. Moderately Severe Disability
	5. Severe Disability
	6. Dead

MONTREAL COGNITIVE ASSESSMENT (MOCA)

**Visuospatial / Executive**	/5
**Naming**	/3
**Attention**	/2
	/1
	/3
**Language**	/2
	/1
**Abstraction**	/2
**Delayed Recall**	/5
**Orientation**	/6
**TOTAL SCORE**	/30

REHABILITATION SESSION ATTENDANCE

Number of missed sessions during each intervention period:

- Intervention period 1: ______________
- Intervention period 2: ______________

CYBERSICKNESS

Number of cybersickness episodes experienced during each intervention period:

- Intervention period 1: ______________
- Intervention period 2: ______________

IDD CODE: ____________ **EVALUATOR:** ______________________ **ASSESSMENT DATE:** ____ /____ /______

ASSESSMENT 1

WISCONSIN GAIT SCALE (WGS)

Assistive device and/or orthosis used: ____________________________

**1. Use of hand-held gait aid***	1	2	3	4	5
**2. Stance time on affected side**	1		2	3	
**3. Step length of the unaffected side**	1		2	3	
**4. Weight shift to the affected side, with or without assistive device**	1		2	3	
**5. Step width**	1		2	3	
**6. Guardedness**	1		2	3	
**7. Hip extension of the affected side**	1		2	3	
**8. External rotation during initial swing**	1		2	3	
**9. Circumduction at mind-swing**	1		2	3	
**10. Hip hiking at mind-swing**	1		3	4	
**11. Knee flexion from toe-off to mind-swing****	1	2	2	3	
**12. Toe clearance**	1		2	3	
**13. Pelvic rotation at terminal swing**	1		2	3	
**14. Initial foot contact**	1		2	3	
**TOTAL SCORE**	**/42**				

SIX-MINUTE WALK TEST (6MWT)

Assistive device and/or orthosis used: ____________________________

- Distance walked in 6 minutes (m): ___________________________

- Were rest breaks required? : _______ ;

If yes, how many? _______ , For how long (s): __________

- Total walking time (s): ______________________
- Total resting time (s): ______________________

REST STOP	TIME (s)
**1ª**	
**2ª**	
**3ª**	
**4ª**	
**5ª**	
**6ª**	
**7ª**	

BERG BALANCE SCALE (BBS)

**1. Sitting to standing**	0	1	2	3	4
**2. Standing unsupported**	0	1	2	3	4
**3. Sitting unsupported with feet supported on the floor or stool**	0	1	2	3	4
**4. Standing to sitting**	0	1	2	3	4
**5. Transfers**	0	1	2	3	4
**6. Standing unsupported with eyes closed**	0	1	2	3	4
**7. Standing unsupported with feel together**	0	1	2	3	4
**8. Reaching forward with outstretched arm while standing**	0	1	2	3	4
**9. Retrieving object from floor while standing**	0	1	2	3	4
**10. Turning to look behind while standing**	0	1	2	3	4
**11. Turning 360 degrees**	0	1	2	3	4
**12. Placing alternate foot on stool while standing unsupported**	0	1	2	3	4
**13. Standing with one foot in front**	0	1	2	3	4
**14. Standing on one foot**	0	1	2	3	4
**TOTAL SCORE**	**/56**				

MODIFIED ASHWORTH SCALE (MAS)

Muscles Assessed: (score 0-5)

**Pectoralis Major**		**Hamstrings**	
**Biceps Brachii**		**Hip Adductors**	
**Finger Flexors**		**Triceps Surae**	

FUGL-MEYER ASSESSMENT LOWE-EXTREMITY (FM-LE)

**E. LOWER EXTREMITY**		**/28**		
**I. Reflex Activity**		/4		
**Flexor**		0	2	
**Extensor**		0	2	
**II. Voluntary Movement Within Synergies**		/14		
**Flexor Sinergy**	**Hip Flexion**	0	1	2
	**Knee Flexion**	0	1	2
	**Ankle Dorsiflexion**	0	1	2
**Extensor Sinergy**	**Hip Extension**	0	1	2
	**Hip Adduction**	0	1	2
	**Knee Extension**	0	1	2
	**Ankle Plantarflexion**	0	1	2
**III. Voluntary Movement Mixing Synergies**		/4		
**Knee Flexion**		0	1	2
**Ankle Dorsiflexion**		0	1	2
**IV. Voluntary Movement with Little or No Synergies**		/4		
**Knee Flexion 90º**		0	1	2
**Ankle Dorsiflexion**		0	1	2
**V. Normal Reflex Activity**		/2		
**Reflex Activity**		0	1	2
**F. COORDINATION / SPEED**		**/6**		
**Tremor**		0	1	2
**Dysmetria**		0	1	2
**Timing**		0	1	2
**TOTAL MOTOR FUNCTION SCORE E – F**		**/34**		

IDD CODE: ____________ **EVALUATOR:** _______________________ **ASSESSMENT DATE:** _____ /_____ /_______

ASSESSMENT 2

WISCONSIN GAIT SCALE (WGS)

Assistive device and/or orthosis used: ______________________________

**1. Use of hand-held gait aid***	1	2	3	4	5
**2. Stance time on affected side**	1		2	3	
**3. Step length of the unaffected side**	1		2	3	
**4. Weight shift to the affected side, with or without assistive device**	1		2	3	
**5. Step width**	1		2	3	
**6. Guardedness**	1		2	3	
**7. Hip extension of the affected side**	1		2	3	
**8. External rotation during initial swing**	1		2	3	
**9. Circumduction at mind-swing**	1		2	3	
**10. Hip hiking at mind-swing**	1		3	4	
**11. Knee flexion from toe-off to mind-swing****	1	2	2	3	
**12. Toe clearance**	1		2	3	
**13. Pelvic rotation at terminal swing**	1		2	3	
**14. Initial foot contact**	1		2	3	
**TOTAL SCORE**	**/42**				

SIX-MINUTE WALK TEST (6MWT)

Assistive device and/or orthosis used: ______________________________

- Distance walked in 6 minutes (m): ___________________________

- Were rest breaks required? : _______ ;

If yes, how many? _______ , For how long (s): ___________

- Total walking time (s): _______________________
- Total resting time (s): _______________________

REST STOP	TIME (s)
**1ª**	
**2ª**	
**3ª**	
**4ª**	
**5ª**	
**6ª**	
**7ª**	

BERG BALANCE SCALE (BBS)

**1. Sitting to standing**	0	1	2	3	4
**2. Standing unsupported**	0	1	2	3	4
**3. Sitting unsupported with feet supported on the floor or stool**	0	1	2	3	4
**4. Standing to sitting**	0	1	2	3	4
**5. Transfers**	0	1	2	3	4
**6. Standing unsupported with eyes closed**	0	1	2	3	4
**7. Standing unsupported with feel together**	0	1	2	3	4
**8. Reaching forward with outstretched arm while standing**	0	1	2	3	4
**9. Retrieving object from floor while standing**	0	1	2	3	4
**10. Turning to look behind while standing**	0	1	2	3	4
**11. Turning 360 degrees**	0	1	2	3	4
**12. Placing alternate foot on stool while standing unsupported**	0	1	2	3	4
**13. Standing with one foot in front**	0	1	2	3	4
**14. Standing on one foot**	0	1	2	3	4
**TOTAL SCORE**	**/56**				

MODIFIED ASHWORTH SCALE (MAS)

Muscles Assessed: (score 0-5)

**Pectoralis Major**		**Hamstrings**	
**Biceps Brachii**		**Hip Adductors**	
**Finger Flexors**		**Triceps Surae**	

PATIENT GLOBAL IMPRESSION OF IMPROVEMENT (PGI-I)

Rate from 1 to 7 how useful the therapy has been for your rehabilitation:

**Completely useful**	**Very useful**	**Useful**	**Somewhat useful**	**Slightly useful**	**Minimally useful**	**Not useful**
1	2	3	4	5	6	7

Rate from 1 to 7 how much your condition has improved with the therapy used:

**Very much improved**	**Moderately improved**	**Slightly improved**	**No change**	**Slightly worse**	**Moderately worse**	**Very much worse**
1	2	3	4	5	6	7

FUGL-MEYER ASSESSMENT LOWE-EXTREMITY (FM-LE)

**E. LOWER EXTREMITY**		**/28**		
**I. Reflex Activity**		/4		
**Flexor**		0	2	
**Extensor**		0	2	
**II. Voluntary Movement Within Synergies**		/14		
**Flexor Sinergy**	**Hip Flexion**	0	1	2
	**Knee Flexion**	0	1	2
	**Ankle Dorsiflexion**	0	1	2
**Extensor Sinergy**	**Hip Extension**	0	1	2
	**Hip Adduction**	0	1	2
	**Knee Extension**	0	1	2
	**Ankle Plantarflexion**	0	1	2
**III. Voluntary Movement Mixing Synergies**		/4		
**Knee Flexion**		0	1	2
**Ankle Dorsiflexion**		0	1	2
**IV. Voluntary Movement with Little or No Synergies**		/4		
**Knee Flexion 90º**		0	1	2
**Ankle Dorsiflexion**		0	1	2
**V. Normal Reflex Activity**		/2		
**Reflex Activity**		0	1	2
**F. COORDINATION / SPEED**		**/6**		
**Tremor**		0	1	2
**Dysmetria**		0	1	2
**Timing**		0	1	2
**TOTAL MOTOR FUNCTION SCORE E – F**		**/34**		

IDD CODE: ____________ **EVALUATOR:** ______________________ **ASSESSMENT DATE:** _____ /_____ /_______

ASSESSMENT 3

WISCONSIN GAIT SCALE (WGS)

Assistive device and/or orthosis used: ____________________________

**1. Use of hand-held gait aid***	1	2	3	4	5
**2. Stance time on affected side**	1		2	3	
**3. Step length of the unaffected side**	1		2	3	
**4. Weight shift to the affected side, with or without assistive device**	1		2	3	
**5. Step width**	1		2	3	
**6. Guardedness**	1		2	3	
**7. Hip extension of the affected side**	1		2	3	
**8. External rotation during initial swing**	1		2	3	
**9. Circumduction at mind-swing**	1		2	3	
**10. Hip hiking at mind-swing**	1		3	4	
**11. Knee flexion from toe-off to mind-swing****	1	2	2	3	
**12. Toe clearance**	1		2	3	
**13. Pelvic rotation at terminal swing**	1		2	3	
**14. Initial foot contact**	1		2	3	
**TOTAL SCORE**	**/42**				

SIX-MINUTE WALK TEST (6MWT)

Assistive device and/or orthosis used: ____________________________

- Distance walked in 6 minutes (m): __________________________

- Were rest breaks required? : _______ ;

If yes, how many? _______ , For how long (s): __________

- Total walking time (s): ______________________
- Total resting time (s): ______________________

REST STOP	TIME (s)
**1ª**	
**2ª**	
**3ª**	
**4ª**	
**5ª**	
**6ª**	
**7ª**	

BERG BALANCE SCALE (BBS)

**1. Sitting to standing**	0	1	2	3	4
**2. Standing unsupported**	0	1	2	3	4
**3. Sitting unsupported with feet supported on the floor or stool**	0	1	2	3	4
**4. Standing to sitting**	0	1	2	3	4
**5. Transfers**	0	1	2	3	4
**6. Standing unsupported with eyes closed**	0	1	2	3	4
**7. Standing unsupported with feel together**	0	1	2	3	4
**8. Reaching forward with outstretched arm while standing**	0	1	2	3	4
**9. Retrieving object from floor while standing**	0	1	2	3	4
**10. Turning to look behind while standing**	0	1	2	3	4
**11. Turning 360 degrees**	0	1	2	3	4
**12. Placing alternate foot on stool while standing unsupported**	0	1	2	3	4
**13. Standing with one foot in front**	0	1	2	3	4
**14. Standing on one foot**	0	1	2	3	4
**TOTAL SCORE**	**/56**				

MODIFIED ASHWORTH SCALE (MAS)

Muscles Assessed: (score 0-5)

**Pectoralis Major**		**Hamstrings**	
**Biceps Brachii**		**Hip Adductors**	
**Finger Flexors**		**Triceps Surae**	

PATIENT GLOBAL IMPRESSION OF IMPROVEMENT (PGI-I)

Rate from 1 to 7 how useful the therapy has been for your rehabilitation:

Completely useful	Very useful	Useful	Somewhat useful	Slightly useful	Minimally useful	Not useful
1	2	3	4	5	6	7

Rate from 1 to 7 how much your condition has improved with the therapy used:

Very much improved	Moderately improved	Slightly improved	No change	Slightly worse	Moderately worse	Very much worse
1	2	3	4	5	6	7

FUGL-MEYER ASSESSMENT LOWE-EXTREMITY (FM-LE)

**E. LOWER EXTREMITY**		**/28**		
**I. Reflex Activity**		/4		
**Flexor**		0	2	
**Extensor**		0	2	
**II. Voluntary Movement Within Synergies**		/14		
**Flexor Sinergy**	**Hip Flexion**	0	1	2
	**Knee Flexion**	0	1	2
	**Ankle Dorsiflexion**	0	1	2
**Extensor Sinergy**	**Hip Extension**	0	1	2
	**Hip Adduction**	0	1	2
	**Knee Extension**	0	1	2
	**Ankle Plantarflexion**	0	1	2
**III. Voluntary Movement Mixing Synergies**		/4		
**Knee Flexion**		0	1	2
**Ankle Dorsiflexion**		0	1	2
**IV. Voluntary Movement with Little or No Synergies**		/4		
**Knee Flexion 90º**		0	1	2
**Ankle Dorsiflexion**		0	1	2
**V. Normal Reflex Activity**		/2		
**Reflex Activity**		0	1	2
**F. COORDINATION / SPEED**		**/6**		
**Tremor**		0	1	2
**Dysmetria**		0	1	2
**Timing**		0	1	2
**TOTAL MOTOR FUNCTION SCORE E – F**		**/34**		
