# Supplementary material for: Effectiveness of dose-matched immersive virtual reality-stationary cycling combined with neurological physiotherapy versus neurological physiotherapy alone in individuals with chronic stroke (CIVIS): A single-blind, randomized crossover trial protocol
Source: PLoS One. 2026 Nov 6;21(11):e0362132. doi: 10.1371/journal.pone.0362132 (PMC13742934; doi:10.1371/journal.pone.0362132)
Supplement: Supplementary file 3 [file pone.0362132.s004.docx]

**Response to Editor (PONE-D-26-24962)**

**Title:** “Efficacy of immersive virtual reality combined with stationary cycling in individuals with stroke (CIVIS): study protocol for a randomized controlled trial."

**New proposed title**: “Effectiveness of dose-matched rehabilitation with immersive virtual reality-stationary cycling combined with neurological physiotherapy versus neurological physiotherapy alone in individuals with chronic stroke (CIVIS): study protocol for a single-blind, randomized crossover trial.”

**GLOBAL RESPONSE**

Dear Editor,

The authors want to thank your thoughtful and constructive comments. All the suggestions were considered and were included into the revised manuscript. Our manuscript has been substantially improved because of the modifications. An itemized point-by-point response to the editor and reviewer’ comments is presented below.

**AA:** Authors

**RV:** Reviewer

**Editor**

The manuscript is suitable in scope as a PLOS ONE Study Protocol and addresses a clinically relevant rehabilitation question. However, it cannot be accepted in its present form because the crossover design, washout justification, primary endpoint definition, sample size rationale, and statistical analysis plan are insufficiently specified. The comparator/intervention dose also requires clarification, and safeguards for blinding, allocation concealment, safety monitoring, and data availability should be strengthened. A substantially revised protocol, preferably with statistical review, should be considered before acceptance.

1. Article type and basic PLOS eligibility

Acceptable in principle. The manuscript is explicitly a randomized controlled trial study protocol, includes the word “protocol” in the title, reports ClinicalTrials.gov registration NCT07529665, and gives a registration date of 2026-04-14. It also states ethics approval from the Andalusian biomedical research ethics committee.

**AA:**

We sincerely thank the editor for the thoughtful and constructive suggestion regarding our manuscript. We appreciate your valuable feedback and comments on our work.

2. Crossover design is the biggest scientific concern. The study uses a 2-period crossover design with a 4-week washout. This is potentially problematic because rehabilitation effects from IVR/cycling are expected to persist; a “washout” may not remove motor-learning or conditioning effects. If carryover exists, the second period is contaminated. The authors do not provide a convincing rationale that 4 weeks is sufficient, nor do they define how carryover, period, or sequence effects will be tested.

**AA:**

We thank the Editor for raising this important concern. We have carefully considered your comments regarding the crossover design and washout period. In this way, the following modifications have been made to address it:

In the "Allocation and randomization" section (page 9, lines 227–237), a detailed justification of the 4-week washout period has been incorporated. The washout duration was selected based on comparable crossover trials of active rehabilitation interventions in stroke populations that have employed similar washout intervals between intervention periods without reporting evidence of carry-over effects [48-50]. We explicitly acknowledge, in line with Patten et al. [50], that carry-over effects are of particular concern in exercise-based interventions where the intent is to induce persistent physiological and motor-learning changes, and that in the worst case, if treatment effects are retained through the washout period, a crossover comparison could simply demonstrate that more therapy is beneficial regardless of type.

To mitigate this risk, the new statistical analysis plan now employs a general linear model (GLM) with treatment, period, sequence, baseline, and center as fixed effects, allowing formal evaluation of carryover, period, and sequence effects prior to the primary efficacy analysis. If a significant sequence effect is observed — indicative of potential carry-over — the treatment effect will be interpreted with caution and a sensitivity analysis restricted to first-period data only will be conducted, following the two-stage approach described by Grizzle [60]. These revisions are described in the "Statistical analysis" section (pages 18-19).

Finally, this concern has also been incorporated as a study limitation in the "Discussion" section (pages 21-22, lines 529–535), where we acknowledge, with reference to Patten et al. [50] and Nolan et al. [61], that despite the washout period adopted and the formal statistical testing of carry-over, the possibility that treatment effects may persist through the washout period cannot be entirely excluded, given the inherent nature of exercise-based interventions designed to induce lasting physiological and motor-learning adaptations. Furthermore, the inadequate reporting and justification of washout periods has been recognized as a frequent methodological limitation in crossover trials [61].

The following references were added:

48. Chen PT, Chiu EC. Reablement of Instrumental Activities of Daily Living for Patients With Stroke: A Randomized Crossover Trial. American Journal of Occupational Therapy. 2024;78. doi:10.5014/AJOT.2024.050288/SUPPL_FILE/SJ-PDF-1-AOT-10.5014_AJOT.2024.050288.PDF

49. Hsieh Y wei, Chang K chou, Hung J wen, Wu C yi, Fu M hui, Chen C chi. Effects of Home-Based Versus Clinic-Based Rehabilitation Combining Mirror Therapy and Task-Specific Training for Patients With Stroke: A Randomized Crossover Trial. Arch Phys Med Rehabil. 2018;99: 2399–2407. doi:10.1016/J.APMR.2018.03.017

50. Patten C, Condliffe EG, Dairaghi CA, Lum PS. Concurrent neuromechanical and functional gains following upper-extremity power training post-stroke. J Neuroeng Rehabil. 2013;10: 1. doi:10.1186/1743-0003-10-1

60. Grizzle JE. The Two-Period Change-Over Design and Its Use in Clinical Trials. Biometrics. 1965;21: 467. doi:10.2307/2528104

61. Nolan SJ, Hambleton I, Dwan K. The Use and Reporting of the Cross-Over Study Design in Clinical Trials and Systematic Reviews: A Systematic Assessment. PLoS One. 2016;11: e0159014. doi:10.1371/JOURNAL.PONE.0159014

3. Primary outcome, sample size, and statistical plan are not adequate yet

The primary outcome is broadly stated as “gait,” assessed by both WGS and 6MWT. They do not clearly specify one primary endpoint, one primary time point, or the exact treatment contrast.

**AA:**

Thank you for your suggestion. Accordingly, in the revised manuscript, a single primary endpoint, a single primary time point, and an explicit treatment contrast have been defined. Gait quality, assessed using the Wisconsin Gait Scale (WGS), has been designated as the sole primary outcome. The primary time point has been specified as the mean change in the WGS total score from baseline to week 4 within each intervention period. The treatment contrast has been defined as the estimated difference in WGS score change between IVR-SC and NP interventions, adjusted for baseline score, period, center, and sequence effects. These specifications can be found in the "Statistical analysis" section (pages 18-19, lines 440-450). All remaining outcomes have been reclassified as secondary in the “Outcomes” section (page 12).

The following reference was added:

46. Julious SA. Sample sizes for clinical trials with normal data. Stat Med. 2004;23: 1921–1986. doi:10.1002/SIM.1783

3. Primary outcome, sample size, and statistical plan are not adequate yet

The sample size is based on WGS and 6MWT estimates, but the final n=24 justification is not fully tied to a crossover model.

**AA:**

We sincerely thank the Editor for this thorough and constructive assessment of the sample size calculation, which has led to a substantial restructuring of this section. We fully agree that the original calculation was insufficiently tied to the crossover model and that basing it on two separate outcomes lacked focus. The revised version addresses these concerns as follows:

The sample size calculation has been entirely revised and is now anchored exclusively to the single primary outcome (WGS), based on the minimal clinically important difference (MCID) of 2.25 points (95% CI [1.75, 2.79]) established by Guzik et al. [45]. The calculation was initially performed using G*Power software for a two-independent-means test (α = 0.05, power = 80%), yielding a minimum of 11 participants per group. This estimate was subsequently recalculated to account for the crossover design by applying an adjustment factor (1 − ρ) for 2×2 crossover trials [46], assuming a conservative within-subject correlation of ρ = 0.5 in the absence of published correlation data for this outcome, yielding a minimum of 6 participants. Accounting for a potential dropout rate of 20%, the multicenter nature of the study, and the heterogeneity inherent to chronic stroke sequelae, a total of 8 participants will be recruited to complete both interventions, including the corresponding washout period.

We explicitly acknowledge that anchoring the target difference to the MCID, rather than to a realistic estimate of the true expected effect, is a recognized limitation of sample size calculations [47]. The calculated sample size should therefore be interpreted as the minimum required to detect a clinically meaningful difference, rather than a guarantee of adequate power for the true underlying effect.

These revisions can be found in the "Sample size" section (page 8).

The following references were used:

45. Guzik A, Drużbicki M, Wolan-Nieroda A, Przysada G, Kwolek A. The Wisconsin gait scale – The minimal clinically important difference. Gait Posture. 2019;68: 453–457. doi:10.1016/J.GAITPOST.2018.12.03650.

46. Julious SA. Sample sizes for clinical trials with normal data. Stat Med. 2004;23: 1921–1986. doi:10.1002/SIM.1783

47. Wong H. Minimum important difference is minimally important in sample size calculations. Trials. 2023;24: 34-. doi:10.1186/S13063-023-07092-8/PEER-REVIEW

3. Primary outcome, sample size, and statistical plan are not adequate yet

More importantly, the planned analysis uses paired t-tests or Wilcoxon signed-rank tests. That is too simple for a crossover RCT because it ignores treatment sequence, period, carryover, baseline values, center effects, and repeated within-person structure. Do not accept without statistical revision. Require a prespecified mixed-effects crossover model, for example treatment, period, sequence, baseline, and center as fixed effects where appropriate, participant as a random effect, and a defined strategy for carryover. Also require a single primary endpoint and primary time point.

**AA:**

We are grateful to the Editor for this rigorous methodological recommendation, which has significantly strengthened the analytical framework of the study. We fully acknowledge that the originally planned paired t-tests and Wilcoxon signed-rank tests were insufficient for a crossover design, as they failed to account for treatment sequence, period, carryover, baseline values, center effects, and repeated within-person structure.

In the revised manuscript, the statistical analysis plan has been substantially restructured. Given the crossover design, a linear mixed-effects model (LMM), or a generalized linear mixed model (GLMM) for non-normally distributed outcomes, will be fitted for each primary, gait quality measured via the WGS, and secondary outcome, with treatment, period, and sequence, entered as fixed effects, together with baseline value and center, and with participant entered as a random effect to account for the repeated within-person structure of the crossover data. Regarding the primary endpoint and time point for primary and secondary outcomes, it will be the mean change in the WGS total score from baseline to week 4 within each intervention period. The treatment contrast will be the estimated difference in WGS score change between IVR-SC and NP interventions, following the standard analysis-of-variance approach recommended for 2×2 crossover designs.

A defined strategy for carryover has been prespecified: a non-significant sequence term will be interpreted as absence of evidence for carryover; if a significant sequence effect is observed, the treatment effect estimate will be interpreted with caution, and a sensitivity analysis restricted to first-period data only will be conducted, following the two-stage approach described by Grizzle [60]. Standardized coefficients (β) derived from the model parameters will be reported as effect size measures.

In addition, according to the editor’ suggestion, an individual responder analysis will be conducted for the WGS.

The new statistical methodology can be found in the "Statistical analysis" section (page 18-19).

The following reference was used:

60. Grizzle JE. The Two-Period Change-Over Design and Its Use in Clinical Trials. Biometrics. 1965;21: 467. doi:10.2307/2528104

4. Intervention/comparator description is inconsistent

The aim says IVR-SC is added to neurological physiotherapy, compared with neurological physiotherapy alone. But the control appears to receive 60 minutes of neurological physiotherapy, while the IVR-SC condition receives 15–30 minutes IVR-SC plus 30 minutes NP. Therefore, the trial is not clearly testing “IVR-SC in addition to NP”; it may be testing partial substitution of NP with IVR-SC

**AA:**

We thank the Editor for this important clarification. We fully agree that the original phrasing "in addition to" could be misleading, as the IVR-SC component does not represent an additional therapy but rather a partial replacement of NP within a dose-matched 60-minute session. To address this, the term "in addition to" has been replaced by "combined with" and “replaced by” throughout the manuscript, including the Title, Abstract section, Objectives (page 7, last paragraph of Introduction section), Intervention description (pages 14-15), and Discussion sections (page 20). The revised framing now accurately reflects the dose-matched design, in which both conditions receive equal total session duration (60 minutes), with the experimental group receiving 30 minutes of IVR-SC combined with 30 minutes of structured NP versus 60 minutes of NP alone.

4. Intervention/comparator description is inconsistent

The intervention is also flexible in duration, intensity, and rest according to participant tolerance. Flexibility is clinically realistic, but the authors need stronger fidelity rules.

**AA:**

We thank the Editor for highlighting the need for stronger fidelity rules. We agree that the original description of the intervention allowed excessive flexibility, which could compromise protocol standardization and replicability. The IVR-SC intervention has been restructured into five sequential blocks with predefined durations, replacing the original time ranges, in the Intervention section (pages 15-17). Target intensity zones have been specified for each block in Table 3. Although block durations are predefined, minor adjustments may be necessary based on individual fatigue levels and functional capacity, given the inherent variability of chronic stroke sequelae.

5. Blinding, allocation concealment, safety, and data availability need strengthening

The principal investigator appears to generate the randomization list, recruit participants, and perform assessments. That weakens assessor blinding and allocation concealment.

**AA:**

We thank the editor for pointing this out and allowing us to clarify this critical methodological aspect. We apologize for the ambiguity in the original text. We have thoroughly updated the "Blinding" section in the revised manuscript.

To ensure rigorous allocation concealment and assessor blinding, tasks have been strictly partitioned among distinct team members who hold independent roles:

Randomization & Allocation Concealment: The randomization sequence is generated and securely maintained by a specific team member (J.A.M-M.) who is not involved in participant recruitment, intervention delivery, or outcome assessment. Clinical supervisors at each center receive the assigned sequence only after baseline assessment to organize treatment delivery. This information was added in the “Allocation and randomization” section (first paragraph, page 9), and Blinding section (second paragraph, page 10).

Participant Recruitment: Clinical supervisors at each center are solely responsible for identifying, screening, and recruiting eligible participants. Treating physiotherapists have access only to the necessary intervention details (Blinding section, second paragraph, page 10).

Assessor Blinding: The outcome assessor (D.A.-P.) works exclusively with coded data, and the master key is accessible only to clinical coordinators. Furthermore, data analysis will be performed using masked treatment labels (A and B) until the dataset is locked (Blinding section, third paragraph, pages 10-11).

Design Justification: In accordance with established methodological recommendations for clinical trials involving immersive technology in neurorehabilitation [41], a single-blind (outcome-assessor-blinded) design is implemented. While participant blinding is unfeasible due to the nature of exercise-based and immersive virtual reality interventions, this inherent limitation does not compromise internal validity as long as outcome assessors remain strictly blinded [41] (Blinding section, first paragraph, page 10).

The following reference was used:

41. Laver KE, Lange B, George S, Deutsch JE, Saposnik G, Chapman M, et al. Virtual reality for stroke rehabilitation. Cochrane Database Syst Rev. 2025;2025: CD008349. doi:10.1002/14651858.CD008349.PUB5

5. Blinding, allocation concealment, safety, and data availability need strengthening

The safety plan is also brief, despite IVR cycling intensities reaching up to 90% HRmax in a stroke population.

**AA:**

We fully agree with the editor that high-intensity exercise in a stroke population warrants a comprehensive and detailed safety protocol. We appreciate this valuable observation, which has helped us strengthen the clinical monitoring and safety plan of our trial. We have expanded the “Safety considerations” section (page 19) in the revised manuscript to explicitly detail our participant safety protocols, stopping criteria, cybersickness monitoring, and emergency procedures as follows:

-Participant heart rate will be continuously monitored using a pulse oximeter to ensure exercise intensity remains strictly within prescribed training zones.

-The session will be immediately pause or terminate if the participant reports chest pain, dyspnea, dizziness, or any other adverse symptoms.

-Symptoms of cybersickness will be systematically recorded after each IVR-SC session using a standardized registry. If significant symptoms persist across more than one consecutive session, the clinical supervisor will evaluate whether to modify or discontinue the intervention.

-Participating centers are equipped with qualified medical personnel and specialized tools to manage potential emergencies or injuries. All adverse events will be formally recorded in the Case Report Form (S3 File) and reported directly to the Principal Investigator.

5. Blinding, allocation concealment, safety, and data availability need strengthening

Finally, the manuscript says datasets “will not be publicly accessible” and only available on reasonable request. PLOS expects Study Protocols to describe where and when data will be made available.

**AA:**

We thank the editor for highlighting this point.

Because this manuscript describes a Study Protocol, no datasets have been generated or analyzed yet. In accordance with PLOS guidelines, we have updated our Data Availability information to state clearly when and how the data will be shared upon completion of the trial. Specifically, we have updated the text as follows (Data collection and data management section, page 12):

"No datasets were generated or analyzed during the current study. All relevant data from this study will be made available upon study completion."

**Reviewer #1:**

**RV:**

Thank you for the opportunity to review manuscript #PONE-D-26-24962 titled “Efficacy of immersive virtual reality combined with stationary cycling in individuals withstroke (CIVIS): study protocol for a randomized controlled trial.” While I think this is a timely and clinically relevant topic to address, I have several comments and suggestions detailed in my specific comments that follow, that I believe will strengthen this protocol. Also, in the Data Availability statement, a brief description of how the data from this study will be made available should be provided. Also, some minor improvement in the scientific writing of this manuscript in the English-language is needed.

SPECIFIC COMMENTS

*Page 1 (Title) – Since this study is comparing two interventions head-to-head with no true control group/period, as well as being a crossover trial, suggest that the title be revised. For example, maybe something like this – “Effectiveness and efficacy of immersive virtual reality combined with stationary cycling in individuals with stroke (CIVIS): study protocol for a single-blind, randomized crossover trial.” I note here that I use the term effectiveness (does the treatment work in the real world?) to align with your proposed intention-to-treat analysis and efficacy (does the treatment work in those who do it?) to align with your per-protocol analysis.

**AA:**

We sincerely thank the reviewer for this thoughtful and constructive suggestion regarding our title. We agree that explicitly specifying the trial design (a single-blind, randomized crossover trial comparing two interventions) significantly improves the clarity and precision of the manuscript title.

Regarding the terminology in the title, we have carefully considered the distinction between effectiveness and efficacy:

- Adoption of "Effectiveness": We fully agree with the reviewer that "effectiveness" is the most appropriate term for our trial, as the intervention is delivered within routine neurorehabilitation centers under conditions reflecting real-world clinical practice, with the primary analysis following an intention-to-treat (ITT) approach.

- Omission of "Efficacy": Rather than framing the study as a dual effectiveness-efficacy trial, we prefer to keep the focus primarily on effectiveness. In our methodological framework, the planned per-protocol (PP) analysis is designed as a prespecified sensitivity analysis to test the robustness of the primary ITT results, rather than a standalone efficacy trial.

Accordingly, we have updated the title of the manuscript (Page 1) to incorporate the recommended study design elements:

Revised Title: Effectiveness of dose-matched rehabilitation with immersive virtual reality-stationary cycling combined with neurological physiotherapy versus neurological physiotherapy alone in individuals with chronic stroke (CIVIS): study protocol for a single-blind, randomized crossover trial.

Following this rationale, we have systematically replaced the term “efficacy” with “effectiveness” throughout the entire manuscript to ensure complete conceptual and terminological consistency.

**RV:**

*Page 2 (Abstract) – In addition to statistical significance, suggest that you tell the reader what the minimal clinically important difference (MCID) will be for changes in gait.

**AA:**

We thank the reviewer for this excellent recommendation. Evaluating clinical relevance alongside statistical significance is indeed crucial for interpreting functional outcomes in stroke rehabilitation.

As suggested, we have updated the Abstract to explicitly define the minimal clinically important difference (MCID) threshold and describe how responder analyses will be incorporated into the study framework.

Specifically, the following statement has been added to the Abstract (Methods section, Page 2):

"An individual responder analysis will be performed to determine if participants exceeded the minimal clinically important difference (MCID) of 2.25 points on the WGS."

**RV:**

*Page 5 (Introduction) – Since you’re talking about cardiorespiratory fitness here, suggest that you exclude “squats” since this is a resistance training versus aerobic exercise that is not typically described in terms of improving cardiorespiratory fitness.

**AA:**

We completely agree with the reviewer’s observation. Squats represent a resistance training exercise rather than an aerobic exercise modality aimed at improving cardiorespiratory fitness, and its inclusion in this context was inappropriate.

As suggested, we have removed the word "squats" from the Introduction section (fifth paragraph, Page 5). The revised text now reads as follows:

"Commonly used exercises include treadmill walking, stationary cycling (SC), and aquatic therapy [19,20]. Among these, SC has received particular attention."

**RV:**

*Page 8 (Participants) – Suggest that you be more specific about whether those with ischemic and/or hemorrhagic stroke will be eligible for inclusion as well as whether men and/or women will be included.

**AA:**

We agree with the reviewer that explicitly stating these criteria improves the clarity and completeness of our participant selection protocol.

As suggested, we have updated the Participants section (Inclusion criteria) to specify that both male and female adults, as well as individuals with either ischemic or hemorrhagic stroke, are eligible for participation.

The revised text in the manuscript (Page 8, Lines [X–Y]) now reads as follows:

"Inclusion criteria include adults aged 18 years or older, both male and female; a medically confirmed diagnosis of chronic ischemic or hemorrhagic stroke (≥6 months)...".

**RV:**

*Page 8 (Sample size) – In my opinion, this is very well done.

**AA:**

We appreciate your valuable feedback and comments on our work.

**RV:**

*Pages 9 and 10 (Blinding) – Since many non-exercise folks are not familiar, I think an additional and more specific emphasis on mentioning that it is virtually impossible to blind participants to the intervention they receive in this type of trial should be made here. While this is an inherent limitation of any study of this nature, it is not through any fault of the investigative team.

**AA:**

We completely agree with the reviewer’s perspective. Explicitly addressing the inability to blind participants in exercise and virtual reality interventions provides essential context for readers outside the field and reinforces the methodological rigor of maintaining outcome-assessor blinding.

As recommended, we have updated the Blinding section (first paragraph, Page 10) to explicitly describe this inherent limitation and its clinical trials framework.

The revised text reads as follows:

"A single-blind, outcome-assessor-blinded design will be implemented, in accordance with methodological recommendations for clinical trials involving immersive technology in neurorehabilitation [41]. Participant blinding is not possible, as participants are necessarily aware of the intervention they receive. This is an inherent limitation of exercise-based and IVR clinical trials and does not reflect any fault of the investigative team. However, this does not compromise the internal validity of the study as long as outcome assessors remain blinded [41]."

The following reference was used:

41. Laver KE, Lange B, George S, Deutsch JE, Saposnik G, Chapman M, et al. Virtual reality for stroke rehabilitation. Cochrane Database Syst Rev. 2025;2025: CD008349. doi:10.1002/14651858.CD008349.PUB5

**RV:**

*Pages 16 and 17 (Statistical analysis) – First, since multiple methods exist, please describe, with appropriate citations, how you plan on performing your intention-to-treat analysis for this randomized crossover trial. Second, please tell the reader if any adjustments will be made for the multiple statistical tests conducted. If not, then this should also be stated. Third, rather than focusing on a generic magnitude of effect such as Cohen’s d, why not focus on the MCID for each of your outcomes? Fourth, suggest that you tell the reader what baseline and sociodemographic variables you will include as well as how multicollinearity will be handled before conducting your multiple linear regression analysis. Fifth, since the interventions will be conducted at different sites, it would seem appropriate to conduct some type of sensitivity analysis by site to see if any differences exist in your outcomes of interest, for example, gait as assessed using the WGS and 6MWT. Finally, what about estimating the number/percentage of responders in your study?

**AA:**

We express our sincere gratitude to the reviewer for these insightful and comprehensive comments. In direct response to this feedback, we have completely overhauled and rewritten our statistical analysis plan from the ground up. The revised Statistical Analysis section (Pages 18-19) now presents an entirely new, updated, and rigorous analytical framework tailored to the trial's design and clinical objectives.

Specifically, the revised section now incorporates:

Intention-to-Treat (ITT) Framework: A detailed description of the primary ITT approach, implemented via a Linear Mixed-Effects Model (LMM / GLMM) suitable for 2×2 crossover designs [46,60], including fixed effects (treatment, period, sequence, baseline values, and center) and participant as a random effect, along with secondary per-protocol sensitivity analyses.

Baseline & Normality Assessments: Standardized reporting of baseline demographic variables and formal normality testing (Shapiro-Wilk) to determine model selection.

Center Control: The explicit inclusion of center in the model to account for potential inter-site variability.

Clinical Relevance & Responder Analysis: An individual responder analysis based on the Minimal Clinically Important Difference (MCID) threshold for gait quality (WGS), reporting both the count and percentage of responders.

We believe these updates significantly enhance the clarity and robustness of our statistical plan.

In addition, the following references were used:

45. Guzik A, Drużbicki M, Wolan-Nieroda A, Przysada G, Kwolek A. The Wisconsin gait scale – The minimal clinically important difference. Gait Posture. 2019;68: 453–457. doi:10.1016/J.GAITPOST.2018.12.036

46. Julious SA. Sample sizes for clinical trials with normal data. Stat Med. 2004;23: 1921–1986. doi:10.1002/SIM.1783

60. Grizzle JE. The Two-Period Change-Over Design and Its Use in Clinical Trials. Biometrics. 1965;21: 467. doi:10.2307/2528104

**RV:**

*Pages 19 and 20 (Discussion) – Another potential limitation that should be mentioned is that the results of the current study may not be generalizable to other similar populations in those with stroke.

**AA:**

We fully agree with the reviewer that the generalizability of our findings is a critical limitation that deserves explicit emphasis in the Discussion section, particularly given the clinical heterogeneity of stroke populations.

As recommended, we have updated the Discussion section (Pages 19–20, Lines [X–Y]) to include this limitation.

The added text reads as follows:

"Finally, findings from this study may not be generalizable to other stroke populations with different clinical profiles, severity levels, or rehabilitation contexts, given the inherent heterogeneity of chronic sequelae [42]. Findings should therefore be interpreted with caution and replicated in larger and more diverse samples."

The following reference was used:

42. Krohn M, Rintala A, Immonen J, Sjögren T. The Effectiveness of Therapeutic Exercise Interventions With Virtual Reality on Balance and Walking Among Persons With Chronic Stroke: Systematic Review, Meta-Analysis, and Meta-Regression of Randomized Controlled Trials. J Med Internet Res. 2024;26. doi:10.2196/59136
