# Supplementary material for: Effectiveness of dose-matched immersive virtual reality-stationary cycling combined with neurological physiotherapy versus neurological physiotherapy alone in individuals with chronic stroke (CIVIS): A single-blind, randomized crossover trial protocol
Source: PLoS One. 2026 Nov 6;21(11):e0362132. doi: 10.1371/journal.pone.0362132 (PMC13742934; doi:10.1371/journal.pone.0362132)
Supplement: Supplementary file 4 [file pone.0362132.s005.docx]

**Response to Editor (PONE-D-26-24962R1)**

**Title:** “Effectiveness of dose-matched immersive virtual reality-stationary cycling combined with neurological physiotherapy versus neurological physiotherapy alone in individuals with chronic stroke (CIVIS): single-blind, randomized crossover trial protocol."

**New proposed title**: “Effectiveness of dose-matched immersive virtual reality-stationary cycling combined with neurological physiotherapy versus neurological physiotherapy alone in individuals with chronic stroke (CIVIS): a single-blind, randomized crossover trial protocol.”

**GLOBAL RESPONSE**

Dear Editor and Reviewer,

The authors want to thank your thoughtful and constructive comments. All the suggestions were considered and were included into the revised manuscript. Our manuscript has been substantially improved because of the modifications. An itemized point-by-point response to the reviewer’ comments is presented below.

**AA:** Authors

**RV:** Reviewer

**Reviewer #1:**

**RV:**

Thank you for the opportunity to review revised manuscript #PONE-D-26-24962R1, now titled “Effectiveness of dose-matched immersive virtual reality-stationary cycling combined with neurological physiotherapy versus neurological physiotherapy alone in

individuals with chronic stroke (CIVIS): single-blind, randomized crossover trial protocol.” In my opinion, the authors have done a very nice job in responding to my previous comments. I only have the following two minor comments, listed below.

SPECIFIC/MINOR COMMENTS

*Page 1 (Title) – In my opinion, this title now reads much better. My only additional suggestion is to insert “A” before the term “single-blind”.

**AA:**

We sincerely thank the reviewer for this suggestion regarding our title. Accordingly, we added “a” before the term “single-blind”.

**RV:**

*Pages 18, lines 441 through 446 (Statistical analysis) – As currently written, this is a long sentence and difficult for me to understand. Therefore, I would suggest that you consider either breaking this up into two or more sentences or writing this more concisely.

**AA:**

We thank the reviewer for this helpful suggestion. We have revised the sentence by splitting it into two shorter, clearer sentences. The text now reads: “*For each primary and secondary outcome, a linear mixed-effects model (LMM) will be fitted for normally distributed outcomes, or a generalized linear mixed model (GLMM) for non-normally distributed outcomes. Treatment, period, sequence, baseline value, and center will be entered as fixed effects, with participant included as a random effect to account for the crossover structure*.”
